# Metal Bioaccumulation by Estuarine Food Webs in New England, USA

**DOI:** 10.3390/jmse4020041

**Published:** 2016-06-03

**Authors:** Celia Y. Chen, Darren M. Ward, Jason J. Williams, Nicholas S. Fisher

**Affiliations:** 1 Department of Biological Sciences, Dartmouth College, Hanover, NH 03755, USA; 2 Department of Fisheries Biology, Humboldt State University, Arcata, CA 95521, USA; darren.ward@humboldt.edu; 3 Department of Civil and Environmental Engineering, Washington State University, Pullman, WA 99164, USA; jason.williams2@email.wsu.edu; 4 School of Marine and Atmospheric Sciences, Stony Brook University, Stony Brook, NY 11794, USA; nicholas.fisher@stonybrook.edu

**Keywords:** metals, estuary, bioaccumulation, AVS-SEM

## Abstract

Evaluating the degree of metal exposure and bioaccumulation in estuarine organisms is important for understanding the fate of metals in estuarine food webs. We investigated the bioaccumulation of Hg, methylmercury (MeHg), Cd, Se, Pb, and As in common intertidal organisms across a watershed urbanization gradient of coastal marsh sites in New England to relate metal exposure and bioaccumulation in fauna to both chemical and ecological factors. In sediments, we measured metal and metalloid concentrations, total organic carbon (TOC) and SEM-AVS (Simultaneously extracted metal-acid volatile sulfides). In five different functional feeding groups of biota, we measured metal concentrations and delta ^15^N and delta ^13^C signatures. Concentrations of Hg and Se in biota for all sites were always greater than sediment concentrations whereas Pb in biota was always lower. There were positive relationships between biota Hg concentrations and sediment concentrations, and between biota MeHg concentrations and both pelagic feeding mode and trophic level. Bioavailability of all metals measured as SEM-AVS or Benthic-Sediment Accumulation Factor was lower in more contaminated sites, likely due to biogeochemical factors related to higher levels of sulfides and organic carbon in the sediments. Our study demonstrates that for most metals and metalloids, bioaccumulation is metal specific and not directly related to sediment concentrations or measures of bioavailability such as AVS-SEM.

## 1. Introduction

Metal contamination is a major global concern in the environment. Metals comprise four of the top ten substances of concern on the Agency for Toxic Substances and Disease Registry 2015 Priority list of Hazardous Substances, with As, Pb, and Hg comprising the top three [1]. Metal contaminants (including metalloids) are common in estuaries where they are often transported from upland watersheds and deposit in estuarine sediments. Metals in sediments can then enter benthic food webs through bioaccumulation in benthic organisms, or enter pelagic food webs after flux into the water column and subsequent bioaccumulation by phytoplankton [2–5]. Identifying variables that control metal bioaccumulation and trophic transfer is therefore important for predicting the effects of metal contamination on estuarine organisms and subsequent human metal exposure through seafood consumption.

Sediment and water concentrations alone do not determine availability or uptake of metals by organisms. Metal bioavailability to ecological receptors is controlled by complex physical, chemical, and biological factors that affect exposure and uptake patterns [6]. These factors include metal speciation (controlled by redox, organic matter, sulfides), metal concentration in aqueous and particulate (food) phases, and ecological processes such as feeding strategies and trophic position of exposed organisms [7,8].

Numerous studies have described factors controlling the bioavailability of metals for aquatic organisms [9,10]. These studies have examined mechanisms of toxicity, kinetic models of uptake, intracellular speciation modeling (Biotic Ligand Models), metal detoxification in aquatic organisms, and bioaccumulation in different body tissues [11–14]. Metal bioaccumulation has also been used as an endpoint for evaluating bioavailability using the tissue residue approach [15–19]. Overall, there are fewer field studies than experimental studies of metal bioaccumulation in coastal food webs, particularly intertidal food webs. The relationship between sediment concentrations and biota concentrations of metals has also not been thoroughly explored, particularly comparing multiple metals across a range of sites that differ in the degree of metal contamination. Estuarine sediments can be sources of metal bioaccumulation in resident food webs and these can serve as vectors of contaminants to human exposure via seafood consumption [20–22].

Here, we investigated bioaccumulation of metal and metalloid contaminants (Hg, MeHg, Se, As, Cd, and Pb) in benthic and pelagic biotic receptors across a gradient of intertidal sites in New England estuaries. The sites we selected encompassed a broad range of watershed urbanization and sediment metal concentrations. All of these metals and metalloids are prevalent at anthropogenically contaminated estuarine sites, but they differ in their routes of uptake and modes of toxicity [10].

Of the trace elements considered here, only Se is biologically essential for many organisms. There are no known biological functions for Hg (in any form), Pb, and As, and the principal use of Cd is as a replacement for Zn as a co-factor for the enzyme carbonic anhydrase [19]. Se is required by a broad number of organisms as an enzyme cofactor, most particularly for the antioxidant, glutathione peroxidase [23]. Because it is biologically essential, the health of organisms may be impaired by insufficient concentrations of Se, but Se can also be acutely toxic when concentrations exceed an optimal level [24]. In contrast, organisms are never stressed by insufficient concentrations of the other trace elements considered here because they are non-essential, but all can be toxic at elevated concentrations, particularly when the sequestering ability of an organism is exceeded by high rates of metal acquisition [25]. Both Se and the non-essential elements considered here (Hg, Cd, Pb, and As) have strong affinities for S and amino functions and consequently are commonly associated with proteins [26].

Generally, the bioavailability of each of these metals and metalloids to estuarine fauna is dependent on the physico-chemical properties of the sediments and species-specific feeding modes, routes of uptake, and assimilation and efflux rates [10,12,13]. In marine ecosystems, Hg, Pb, As, Cd, and Se are enriched in sediments relative to the water column. Of these elements, the binding of Cd to particulate matter is most affected by salinity, and its particle affinity (hence, binding strength to sediments) is inversely related to salinity due to chloro-complexation [27]. Hg, MeHg, its most bioavailable form, and Se are metals that are highly assimilated, bioaccumulated through diet in marine organisms, and known to biomagnify in marine food webs [13,28]. Moreover, Hg and Se in sediments can form mercuric selenide which has been shown reduce the bioavailability of Hg for methylation into the more toxic form, MeHg [29], Arsenic, Cd and Pb, in contrast to Hg and Se, have lower assimilation efficiencies and have been shown to biodiminish in aquatic food webs [13,30–32].

The overarching goal of this study was to investigate the relationship between metal and metalloid contamination in sediments and bioaccumulation in intertidal food webs across a range of diverse metals to evaluate larger-scale patterns across sites. Specifically, we determined if sediment concentrations of metals and metalloids, organic carbon concentrations, or measures of element bioavailability and bioaccumulation were predictive of concentrations in a variety of estuarine fauna. We focused on metal and metalloid concentrations in lower trophic level organisms, because these taxa link these elements in sediments and the water column to pelagic fisheries, which are important sources of human exposure to metal contaminants. At each site, we collected animals from a range of feeding groups and trophic levels, including filter feeders (blue mussels and ribbed mussels; *Mytilus edulis* and *Geukensia demissa*), detritivores (amphipods), and omnivorous fish and invertebrates (killifish, *Fundulus heteroclitus*; green crabs, *Carcinus maenas*; and shrimp, *Paleomonetes pugio, Crangon septemspinosus*). All of these lower trophic level species are also resident species that have strong site fidelity and small ranges such that their metal burdens reflect exposures in the areas from which they were sampled [33–35]. In order to investigate the relationships between sediment and biotic metals, we compared tissue concentrations of metals in estuarine organisms to sediment concentrations (both Total Organic Carbon (TOC), normalized and non-normalized) and to simultaneously extracted metal (SEM) for Cd, Hg, and Pb. We also evaluated metal bioavailability by measuring SEM-AVS and by calculating Biota Sediment Accumulation Factors (BSAF). Finally, we also examined the relationship of food source and trophic position to bioaccumulation for each metal.

## 2. Materials and Methods

### 2.1. Field Sites

**Figure 1:**
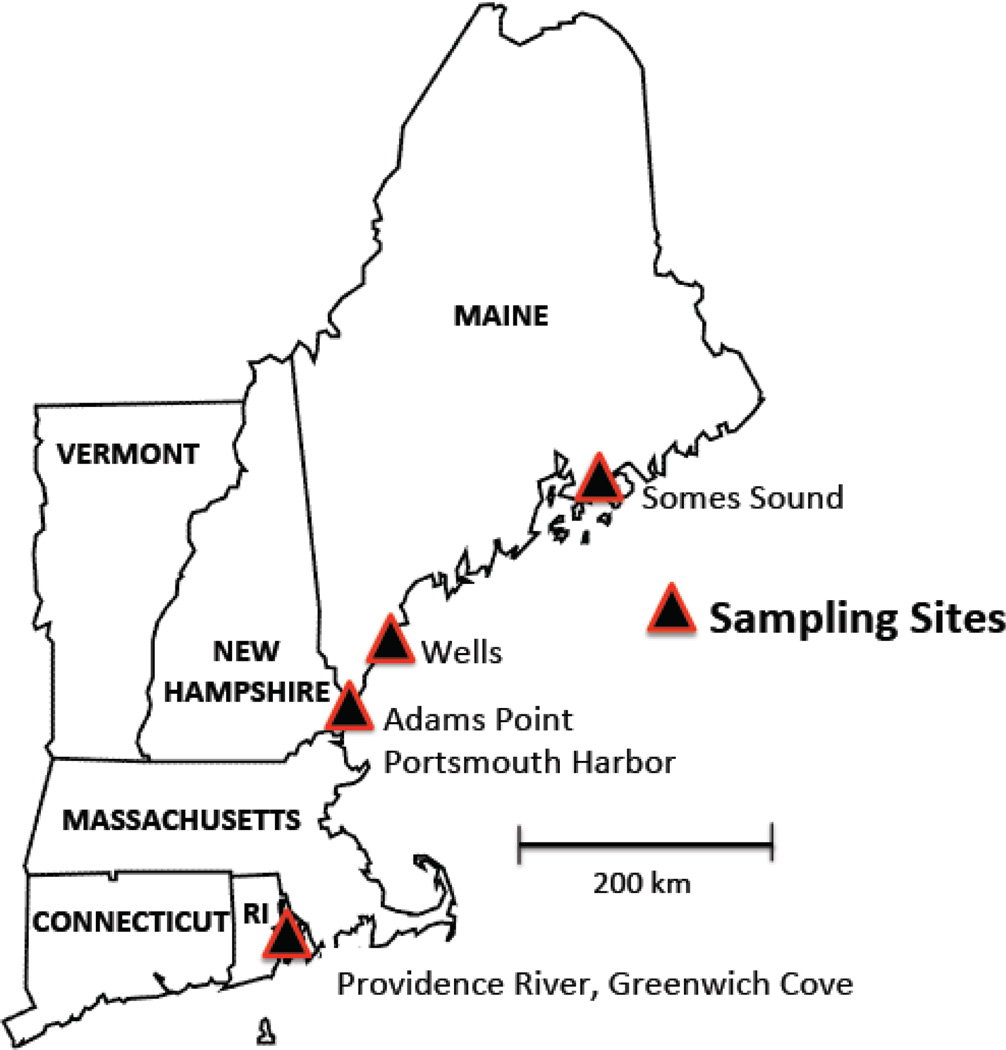
New England field sites. Six estuarine field sites in Maine (Mount Desert Island, Wells), New Hampshire (Portsmouth Harbor, Adams Point), and Rhode Island (Bold Point, Greenwich Cove).

We studied six sites that encompassed a broad range of watershed land use and urbanization. Four sites were located in the Gulf of Maine, and two sites in Narragansett Bay, RI (Figure 1). The sites included: (1) Adams Point in southeast Great Bay NH, where direct industrial inputs are low [36] and land use is relatively forested; (2) the Portsmouth Harbor Region of Great Bay, which is highly industrialized and adjacent to numerous contaminated sites; (3) the Webhannet Estuary in Wells ME, which is undeveloped except for some residential areas (mostly seasonal); (4) Somes Sound on Mount Desert Island Maine adjacent to Acadia National Park, which is undeveloped but receives relatively high atmospheric inputs of Hg [37]; (5) Greenwich Cove, a residentially developed site with a large boat marina on the eastern side of Narragansett Bay and (6) Providence River Estuary a highly industrialized site and shipping channel at the head of Narragansett Bay. At Portsmouth Harbor, the proximity to Hg contaminated sites at the adjacent Portsmouth Naval shipyard are previously documented [38]. At all sites, we sampled intertidal areas with similar patterns of tidal inundation: sediment samples were taken at low tide in 0.5 m depth of water and biotic samples were collected at both low tide (invertebrates) and mid tide via seining. Salinities were taken at high tide at each site and ranged from 30 to 32 ppt in all sites but the upper Webhannet Estuary and the Providence River Estuary where salinities were 24–25 ppt.

### 2.2. Sediment Samples

Sediment samples were collected in summer 2006 at each site using a 6 cm diameter coring tube. The top 2 cm of nine sediment cores taken in an area of approximately 100 m^2^ were composited into a single sample. Aliquots of the composite were freeze-dried, homogenized, and analyzed for total Hg, MeHg, Se, Cd, As, and Pb and total organic carbon as described below. Separate sediment samples (three replicates per site) were collected for SEM-AVS analysis by taking a 4 cm plug of sediment using a 250 mL glass jar under water to prevent contact with air and freezing the sample immediately.

### 2.3. SEM-AVS and TOC Analysis of Sediments

SEM-AVS samples were shipped to Battelle Marine Sciences Laboratory where they were extracted and analyzed for AVS/SEM in accordance with Battelle SOP MSL-C-001. Sulfide was converted to hydrogen sulfide, which was purged from the sample, converted to methylene blue, and measured on a spectrometer. SEM extracts were analyzed for total Hg by Cold Vapor Atomic Fluorescence (CVAF) in accordance with Battelle SOP MSL-I-013 based on EPA Method 1631 Revision E, and by Inductively Coupled Plasma-Mass Spectrometry (ICP-MS) for all other extracts (Cd, Cu, Hg, Ni, Pb, and Zn) in accordance with Battelle SOP MSL-I-022 adapted from USEPA Method 1638. SEM-AVS was calculated by summing the SEM values for all six metals (Cd, Cu, Hg, Ni, Pb, and Zn) and subtracting AVS values.

TOC was analyzed using thermal partitioning at 550 °C (EPA 440.0). Total (organic + inorganic) C was determined at 1350 °C combustion temperature. A second sample was combusted at 550 °C to burn off organic C but leaving inorganic C. The residue from that procedure was put through the combustion analyzer at 1350 °C to measure inorganic C. Organic C was calculated as the difference between the two determinations.

### 2.4. Biota samples

We measured Hg (inorganic and MeHg), Se, As, Cd, and Pb in resident benthic and pelagic fish and invertebrates. Invertebrates were sampled using plastic trowels, minnow traps, D nets, pitfall traps, collected by hand, or collected by sieving sediments through a 0.5 mm nylon coated mesh. Animals were not depurated because we wanted to measure whole organisms that are consumed by predators. All collected invertebrates were returned to the lab where they were sorted the same day and identified to the lowest practical taxon which was generally the order or family associated with the functional feeding group of the organism. All samples were handled with trace metal clean techniques and stored in either acid cleaned plastic bags or acid cleaned Teflon vials (for smaller organisms) and frozen. Later, frozen samples were thawed, rinsed with ultra-clean water, weighed, and freeze-dried and homogenized prior to metal analysis. Mussels were removed from their shells prior to freeze-drying. Amphipods collected at each site were pooled in order to obtain at least 3 samples with enough dry weight for metal analysis.

Fish sampling was conducted at mid to high tide levels using fish seines, fyke nets, and minnow traps. Fish were handled and euthanized with protocols approved by the Dartmouth College IACUC (Institutional Animal Care and Use Committee). Fish total lengths and wet weights were measured. For each fish and invertebrate species, we selected similar-sized individuals at all sites for metal to reduce the influence of size on feeding habits and trophic position measured by stable isotopes. However, this procedure did not take into account differences in age of the fish. Fish were frozen in acid rinsed plastic bags for storage and processed in the lab according to Chen *et al.*, 2009 [39].

### 2.5. Metal Analysis of Biota and Sediment Samples

All sample metal analyses (Hg, MeHg, Se, As, Cd, Pb,) other than SEM-AVS were conducted by the Dartmouth Trace Element Core Facility using a magnetic sector inductively coupled plasma-mass spectrometer (ICP-MS ELEMENT2, Thermo-Finnigan, Waltham, MA, USA). Biota samples were analyzed for Hg speciation using isotope dilution gas chromatography-ICPMS. Samples were freeze-dried and homogenized, spiked with an appropriate amount of enriched inorganic ^199^Hg (HgI) and enriched methyl^201^Hg (MeHg) and then extracted in 2–3 mL of KOH/methanol (25% *w/v*), ethylated and analyzed using purge and trap GC-ICP-MS. One of two methods for Hg speciation was employed depending on the expected level of Hg in the original sample which was a function of the initial available sample mass. For samples <20 mg, the methodology involved purging with inert gas and trapping on a Tenax trap which was thermally desorbed and Hg species were quantified by isotope dilution GC-ICP-MS using a high sensitivity Element2 ICP-MS in low resolution mode. For samples >20 mg, samples were analyzed according to previously published methods [40]. The latter methodology is less time-consuming than the purge and trap method, but has higher detection limits and is only suitable for larger initial sample masses. Quality control for MeHg in biota samples was conducted through the analysis of two SRM’s: NIST 2976, mussel tissue with MeHg certified at 0.0278 ± 1.1 µg· g^−1^ and CRC (Ottawa, ON, Canada) DORM-2, dogfish muscle, MeHg concentration of 4.47 ± 0.32 µg· g^−1^. Average recovery for MeHg in DORM-2 was 108% (*n* = 13, r.s.d. = 3.4%) and for NIST 2976 average recovery was 114% (*n* = 12, r.s.d. = 10%). Method detection limits for MeHg analysis by isooctane extraction and capillary GC-ICP-MS (Agilent 7500c, Palo Alto, CA, USA) are 5 ng· g^−1^ assuming an initial sample mass of 200 mg. For the purge and trap GC-ICP-MS (Element 2, Thermo-Fisher, Bremen, Germany) method detection limits are 0.2 ng· g^−1^ based on an initial sample weight of 25 mg.

Tissue and sediment samples for total Hg, As, Se, Cd, and Pb were acid digested with HNO_3_ using a MARSxpress microwave digestion unit (CEM, Matthews, NC, USA). Approximately 100 mg of sample was weighed into a Teflon digestion vessel and 2 mL of Optima HNO_3_ was added. The vessel was heated to 180 °C with a 10 min ramp and 10 min hold. After digestion the sample was brought up to 25 mL volume with deionized water. Total metals were analyzed by inductively coupled plasma mass spectrometry (ICP-MS, 7500cx, Agilent, Santa Clara, CA, USA) using both collision cell and normal mode following the EPA 6020 protocol. The digestion quality control included blank, duplicates and certified reference materials for biotic samples (SRM’s: NIST SRM 2976 mussel tissue *n* = 3, and TORT, NRC-CNRC Canada). Average metal recovery rates for mussel and TORT, respectively, were: THg 114.7 + 12.7, 99.6%; Cd 105 + 2.5, 110%; Pb 110.8 + 6.0, 129%; As 118.5 + 9.0, 106%; Se 114.6 + 13.6, 108.6%). Detection limits based on a 40 mg sample were: THg 0.015 mg/kg: Cd 0.158 mg/kg, Pb 0.016 mg/kg; As 0.128 mg/kg; Se 0.345 mg/kg. Quality control for sediments samples was conducted through analysis to the marine sediment SRM, IAEA-433. Average metal recovery rates were As 80%, Se 90%, Cd 80%, Pb 107%, Zn 87%, Hg 120%. These values all fell within the acceptable range according to EPA QC criteria (75%–125%).

### 2.6. Stable Isotope Analysis

Whole fish tissue, whole invertebrates, and mussels without shells sampled for metals were also analyzed for stable isotopes at the Colorado Plateau Stable Isotope Laboratory. Once samples (3 replicate individuals per species per site) for metal analysis were freeze-dried and homogenized, subsamples of each sample were taken for stable isotope analysis. Approximately 1 mg of homogenous powder of organisms was analyzed for stable isotope ratios (^13^C/^12^C, ^15^N/^14^N). ^13^C was used to identify food sources [41] such as benthic *vs.* pelagic production [42] or marsh plants *versus* phytoplankton [38]. ^15^N was used to identify the relative trophic levels of the organisms within each site [41].

### 2.7. Data Analysis

Because the same mussel, shrimp, and amphipod species were not collected at all sites, biotic data were pooled into five general taxonomic groups for data analysis: amphipod, crab, *Fundulus*, mussel, and shrimp. Previous studies have shown that animals within an animal/functional feeding type process metals similarly (for different predatory teleost species, for example: [28]; for different filter-feeding mytillid mussel species: [43]). Therefore, no attempt was made during data analysis to separate different species in this study. For example, all mussels were pooled together, all amphipods were pooled together, *etc*.

The coefficient of variation of metal concentrations in sediments and for each taxonomic group was calculated for each metal to determine whether the variation in biotic compartments across sites was comparable to variation across sites for sediments. Biota-Sediment Accumulation Factors (BSAFs) were calculated for each species group or metal per site as BSAF = metal concentration in organism (ng· g^−1^ dry wt.)/metal concentration in sediment (ng· g^−1^ dry wt.).

We used general linear models (analysis of covariance, ANCOVA) to evaluate the relationship between sediment characteristics at each site and element concentrations in organisms. The response variable was the mean log_10_-transformed element concentration for each taxonomic group at each site, with separate analyses for each element. We accounted for variation in metal concentrations among taxonomic groups by including taxa as a nominal term in all models. We considered sediment element concentration (log-transformed), TOC-normalized sediment element concentration, sediment TOC, SEM-AVS, and element SEM (Cd, Hg, and Pb) as predictor variables, with a single continuous predictor in each model. Sediments were characterized at the site level, not independently for each taxonomic group at each site, so we conservatively took the number of sites as the degrees of freedom for the continuous predictors.

We used general linear models (GLM) to evaluate the relationship between element concentrations in organisms and two food web variables, trophic level (as indexed by ^15^N) and pelagic feeding (as indexed by ^13^C). The response variable was the mean log_10_-transformed element concentration for each species at each site, with a separate analysis for each element. We accounted for site-to-site variation in metal concentrations and isotopic baselines by including site as a nominal term in the models. This approach assumes that, within each site, ^15^N is linearly related to trophic position [41] and ^13^C is linearly related to the relative proportion of pelagic resources in the diet [44].

## 3. Results

### 3.1. Sediment and Biotic Metals

**Figure 2:**
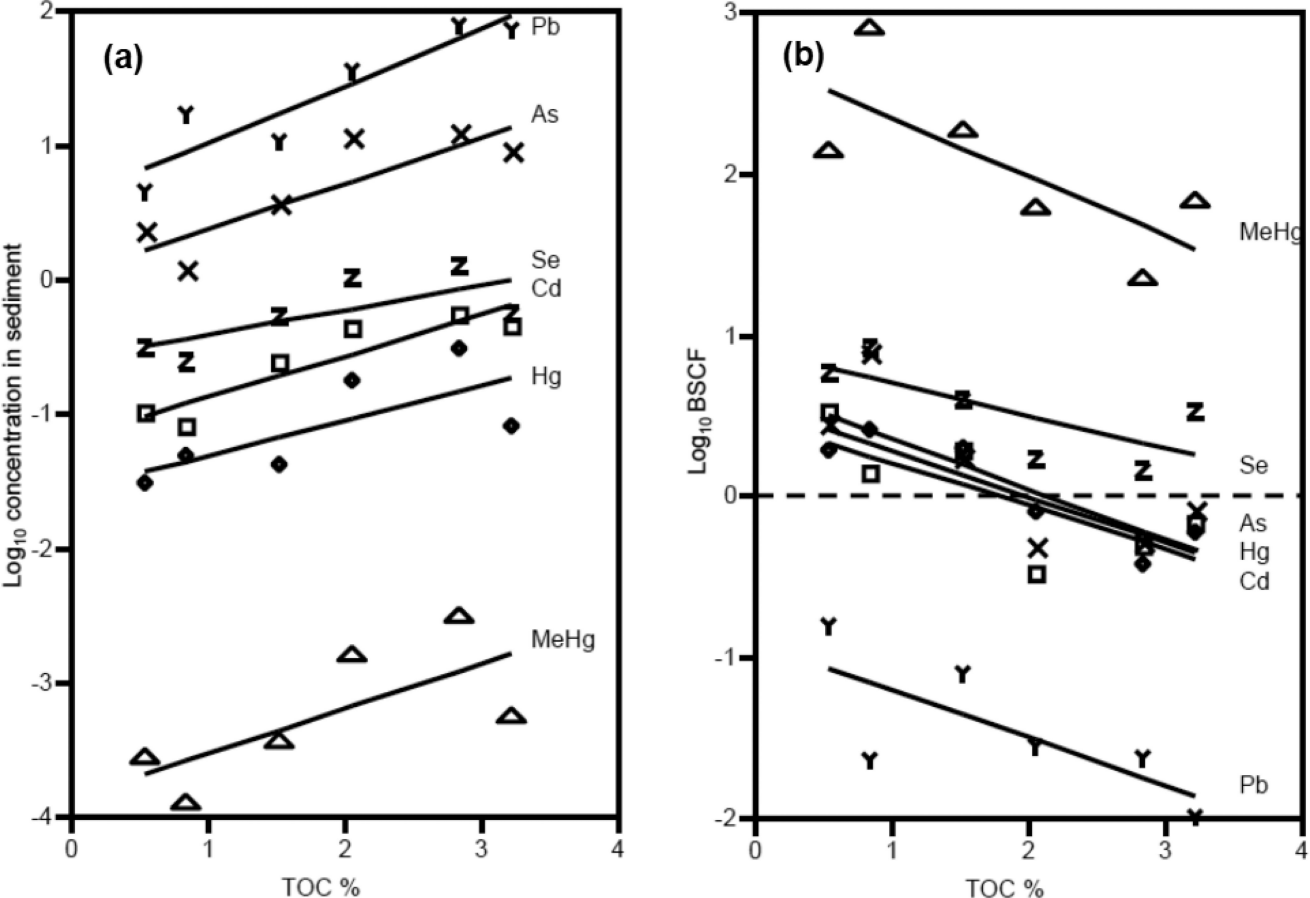
Sediment and biotic metals *vs.* TOC. Relationship of TOC to: (**a**) metal and metalloid concentrations in sediments (log_10_ concentration) and (**b**) Mean Benthic Sediment Concentration Factor (log_10_ BSAF), averaged across all species at each site. Each metal and metalloid is represented by a different symbol: triangle = MeHg, Z = Se, X = As, cross = Hg, square = Cd, and Y = Pb.

**Figure 3:**
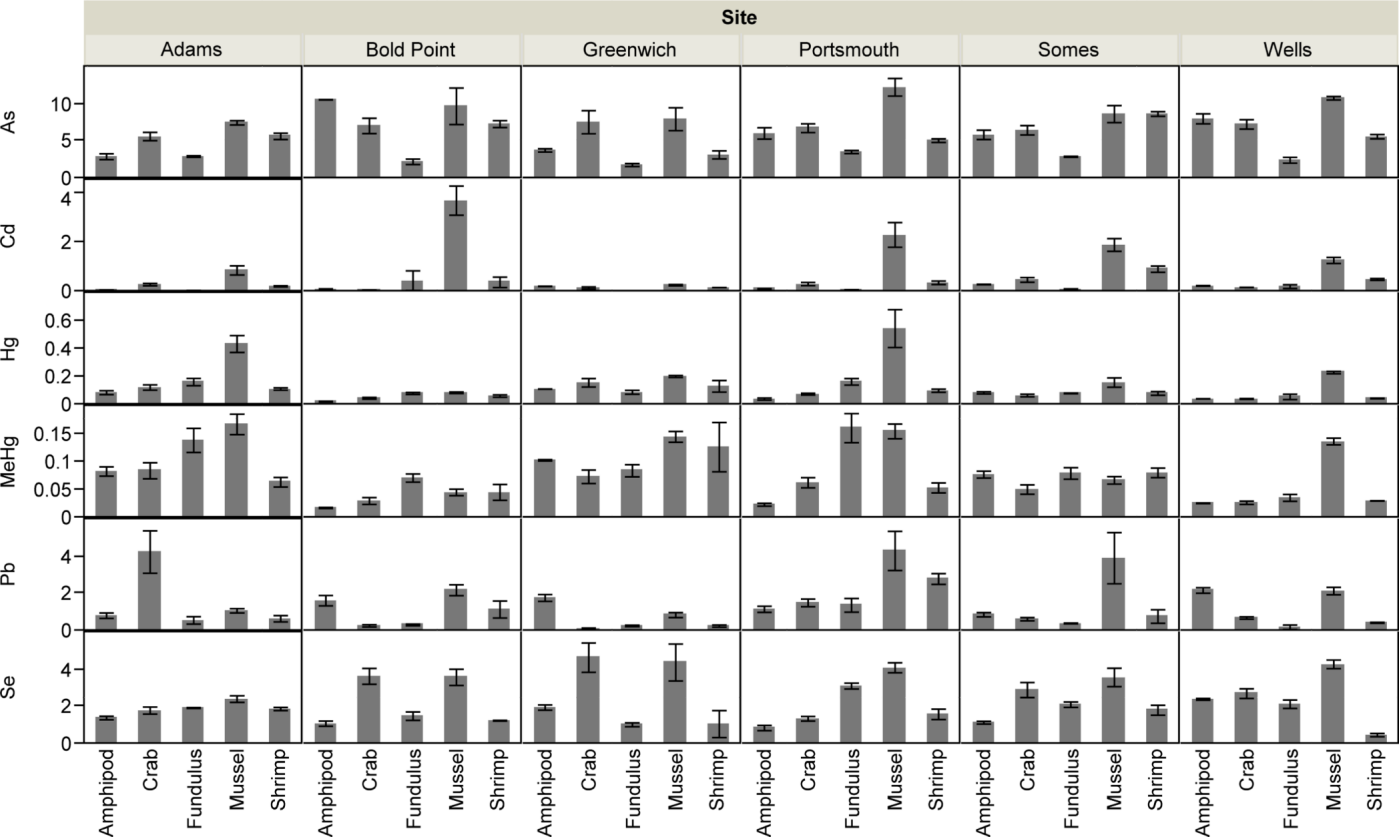
Biotic metal and metalloid concentrations. Element concentrations (ng· g^−1^ dry wt.) for all sites and taxa, reported as mean ± SE.

Sediment metal concentrations ranged widely across sites (Table 1). Across all metals, concentrations in sediments increased with the %TOC (Figure 2a). The SEM-AVS values at all sites were negative, indicating that all metals (except As and Se which do not form insoluble sulfides) were complexed by AVS and considered not bioavailable to benthic organisms (Table 1, Table S1). Moreover, the molar Se:Hg ratio for sediments was >1.0 (ranging from 11.0 to 31.8) across all sites indicating that there was sufficient Se to bind Hg in the sediments [22,29]. There was variation in metal concentrations across different taxonomic groups within sites, which was comparable to the differences between sites (Figure 3). In addition, the CV for sediments across sites was much greater than variation in biota concentrations for Hg, MeHg, and Se, but both less than and greater than the CV for biota concentrations of Cd, As, and Pb which were much more variable across taxa (Table 2).

### 3.2. Biotic Metal Concentration Predictors

Table 3 shows the results of the ANCOVA testing the relationship between response variables (metal concentrations in biota) and predictor variables (sediment metal concentrations, TOC, TOC-normalized sediment metal concentrations). Sediment concentrations were predictive of biotic concentrations for only TOC-normalized Hg concentrations (Table 3, *p* = 0.018). SEM concentrations for Pb were also marginally predictive of biota concentrations (Table 3). Trace metal concentrations in organisms were significantly different across sites and species (Figure 3, Table S1). There was an interaction between site and species, such that no site had elevated trace metal concentrations for all species. Unlike the other metals, sites did not differ significantly in terms of their Se concentrations in biota.

Ecological measures (^13^C and ^15^N) were predictive of biota Hg and MeHg concentrations but not for the other metals (Table 4). Of the five taxonomic groups examined, *Fundulus* occupied the highest trophic level and amphipods the lowest (Table S2 Figure S1). MeHg and Hg concentrations were highest in organisms that were more depleted in ^13^C, indicating that pelagic food sources resulted in higher metal bioaccumulation than benthic food sources. Mussels had the most pelagic signature, reflecting their phytoplankton food source, possibly accounting for their consistently high MeHg concentrations. Higher trophic level organisms, as revealed by ^15^N enrichment, also had higher MeHg concentrations and higher percent of total Hg as MeHg.

Log_10_ BSAFs, reflecting metal bioaccumulation for each animal relative to sediment concentrations at each site, ranged from −0.60 to 1.82 (As), −1.19 to 1.09 (Cd), −0.91 to 0.86 (Hg), 0.87 to 3.05 (MeHg), −2.464 to −0.33 (Pb), and −0.18 to 1.26 (Se) (Figure 2b, mean values presented). Log_10_ BSAF values greater than zero indicated that organisms concentrated metals to levels greater than the sediment from which they came. All the log_10_ BSAFs for MeHg were >0 and all were <0 for Pb, but were both negative and positive for the other metals depending on TOC. In the case of Se, only one species type at one site had a negative Log_10_ BSAF (amphipods in Portsmouth, Great Bay, NH, USA). Across sites, BSAFs were generally higher for the three less urbanized contaminated sites (Wells, ME, USA, Somes Sound, ME, USA and Greenwich, RI, USA) than more urbanized and contaminated sites (Adams Point and Portsmouth in Great Bay, NH, USA, Bold Point in Providence, RI, USA). For all metals, BSAFs were inversely related to sediment TOC and BSAFs for As, Hg, MeHg, and Se were positively related to SEM-AVS (Figure 2b, Table 3).

## 4. Discussion

Understanding the relationship of metals in sediments to bioaccumulation in aquatic organisms is important to understanding the fate of metals in coastal ecosystems from pristine to contaminated. Sediment and porewater metal concentrations are considered to be important routes of exposure in coastal food webs [7]. However, studies vary in their findings about whether bulk sediment concentrations of different metals directly relate to biotic concentrations [5,12,45]. For example, earlier studies of estuarine bivalves and polychaetes showed significant positive relationships between tissue concentrations and acid-extracted sediment concentrations for As, Hg, and Pb [12]. For As and Pb, Fe normalized acid-extracted sediment concentrations were also positively related to tissue concentrations [12]. In this study, we find that bulk sediment concentrations and SEM are only predictive of total Hg across organismal groups. Sediment Hg as a predictor and source of biotic concentrations has been observed in some earlier studies as well [20,46].

### 4.1. Metal Bioavailability

For all metals (Hg, MeHg, As, Cd, Se, Pb), sediment concentrations were positively correlated with sediment TOC, but negatively related to biota BSAF. Positive relationships between TOC and sediment metal concentrations are also well documented in other datasets (USEPA EMAP-NCA; [46]) and occur because dissolved metals readily bind to organic carbon. The inverse correlation of BSAF with TOC is a commonly found relationship and largely due to the positive relationship of sediment concentrations (used as the denominator in the calculation of BSAF) with TOC. This inverse relationship between sediment TOC and BSAF likely occurred because TOC is one of several sediment characteristics that mediates bioavailability [39,47,48]; metal binding organic carbon in sediments limits bioavailability through both aqueous and dietary bioaccumulation pathways, and therefore reduces BSAF values. Thus, sediments enriched in TOC may have stronger binding for the metals than low-TOC sediments, and hence the metals in high TOC sediments may be harder to acquire by organisms, leading to lower accumulation. However, it is only for Hg that there is both a negative relationship between BSAF and TOC, and positive relationship between sediment and biota concentrations.

Our data show that variation in sediment concentrations relative to biota concentrations differed greatly between metals. For Cd, As, and Pb, sediment variation and variation in biota concentrations are comparable. In contrast, total Hg, MeHg, and Se concentrations in sediments vary across sites to a much greater degree than concentrations in biota. Between metals, there are also differences in whether organisms bioaccumulate more or less metal than their associated sediments. For example, MeHg and Se BSAFs for all taxonomic groups are greater than 1.0 indicating that biota concentrations are all higher than sediment concentrations. This may be due to higher assimilation efficiencies and/or greater retention of MeHg and Se for a variety of taxonomic groups [28,32]. This contrasts with low assimilation efficiencies and higher excretion of Pb which bioaccumulates in organisms to much lower levels than concentrations in sediments [49].

The differences in both variation and magnitude of metal concentrations in sediment *vs.* biota may be due to the differences in the feeding behaviors of different organisms and the exposure routes of different metals. In fish, Cd is known to be taken up from the water column via the gills [19], from sediments via porewater ([50], and especially from food [28]. Pb uptake in filter feeding bivalves is via ingestion of particulates [51] and ingestion of sediments by deposit feeding worms [52]. Total Hg, MeHg, and Se metals are taken up predominantly through trophic transfer from food and this likely influences the bioavailability of sediment metal to estuarine herbivores and omnivores [53–56]. Lastly, tissue residue-based approaches for determining thresholds of effect in aquatic organisms have been proposed for MeHg and Se but not for other metals [57].

In numerous studies, SEM-AVS has been demonstrated to be useful in determining toxicity to benthic organisms for a variety of transition metals [58,59]. Toxicity occurs when SEM-AVS > 0. However, other studies show that it does not provide a good measure of bioavailability as related to bioaccumulation [60,61] and organisms can still accumulate metals when SEM-AVS < 0. The negative SEM-AVS values in this study indicate that Cd and Pb should all be complexed with AVS and therefore, not bioavailable. However, both metals are bioavailable given the measureable and in some cases, elevated concentrations in biota and most particularly, the benthic fauna (amphipods and crabs). SEM-AVS is based on exposure via porewater concentration and does not account for ingestion of metals in solid phase even though it is known to be an important pathway (reviewed in Wang and Fisher [62] and Hare *et al.* [63]. Therefore, SEM-AVS can underestimate bioaccumulation and toxicity [60]. In this study, metals were bioaccumulated even in sediments in which SEM-AVS values were always negative, suggesting that metals were either still bioavailable and not all bound to AVS [48], that AVS-bound metals were assimilable [60], or that the exposure of these organisms to metals is not entirely from porewater concentrations in sediments, which is likely the case. The organisms examined in this study were largely epifaunal or pelagic and most likely not as influenced by porewater concentrations.

Although the bioavailability of metals is greatly affected by the concentration of TOC, only Hg in biota was marginally related to carbon normalized sediment concentrations, indicating that sediment Hg concentration may be a predictor for Hg bioaccumulation. A relationship between sediment and tissue concentrations was also shown in past studies of total Hg in estuarine fauna and organic matter normalized concentrations of Hg in sediments [9,20]. While there are relationships of carbon normalized Hg in sediments and biota in this study, the relationship does not hold true for MeHg, the most bioavailable and toxic form. This is consistent with our recent studies which indicate that MeHg in estuarine fish (killifish and Atlantic silversides) is predicted by MeHg in water column particulates, not sediments, and perhaps not surprisingly, sediment MeHg only predicts concentrations in worms [46]. Moreover, sediment concentrations of MeHg are not related to water or particulate concentrations, which are important non-sediment routes of exposure [64]. For the other metals, bulk sediment concentrations (carbon normalized or not) also appear to be unrelated to bioaccumulation.

### 4.2. Ecological Factors and Metal Bioaccumulation

In our study, measurements of stable isotope signatures of food web organisms showed relationships between food sources and metal bioaccumulation for only Hg and MeHg, as seen previously [39,46]. In all cases, organisms deriving their food from pelagic sources (more depleted in ^13^C) had higher Hg and MeHg concentrations than those feeding on less depleted sources. This is similar to results of earlier studies in estuaries and in freshwater systems [39,46,65]. Moreover, organisms with more enriched delta ^15^N also had higher MeHg concentrations and percent of total Hg as MeHg (%MeHg). This has been shown for other food webs in marine and freshwater ecosystems [39,56,66–70] Although not significant, there was also a trend of decreasing Pb concentration with increasing trophic level that has been shown in freshwater studies [31]. This is likely a function of low assimilation of Pb and its tendency to biodiminish with increasing trophic level [49].

## 5. Conclusions

Higher metal concentrations in sediments do not result in higher concentrations of metals in benthic and pelagic organisms across these systems except for total Hg. However, across all sites, BSAFs indicate that organisms consistently bioaccumulate MeHg, and Se to higher concentrations than in sediment, whereas for Pb, concentrations in biota are always lower than sediment concentrations. All SEM-AVS values are negative indicating that metals should all be bound to sediments and not available in porewater yet bioaccumulation still occurs, suggesting the importance of dietary sources. Finally, Hg and MeHg are unique among the metals studied here in their relationship to food web variables; as has been seen in other studies, Hg bioaccumulation is greater in organisms deriving their food from pelagic sources and in higher trophic level organisms. While sediments are repositories for metal contaminants and in most cases managed in remediation, metal concentrations in sediments are not necessarily linked directly to bioaccumulation in benthic and pelagic organisms. Moreover, standard measures of bioavailability, such as SEM-AVS, can indicate the lack of bioavailability even when bioaccumulation is evident. These findings should be considered when using these abiotic measures to infer exposure and uptake by estuarine organisms.

## Supplementary Material

Erratum

## Figures and Tables

**Table 1:** Sediment attributes across six sites in the Gulf of Maine and Narragansett Bay.

Site	As (µg · g ^−1^)	Cd (µg · g ^−1^)	Hg (µg · g ^−1^)	MeHg (µg · g ^−1^)	Pb (µg · g ^−1^)	Se (µg · g ^−1^)	TOC (%)	SEM-AVS
Adams Pt. NH	11.2	0.4	0.2	0.002	37.3	1.1	2.1	−11.7
Bold Point RI	8.8	0.5	0.08	0.0006	75.9	0.6	3.2	−28.6
Greenwich RI	1.2	0.08	0.05	0.0001	17.4	0.3	0.8	−1
MDI ME	3.6	0.2	0.04	0.0004	11.1	0.5	1.6	−14.1
Portsmouth Harbor NH	12.1	0.6	0.3	0.003	79.6	1.3	2.8	−28
Wells ME	2.2	0.1	0.03	0.0003	4.7	0.3	0.5	−15.2

**Table 2:** Variation in sediment and biotic metal and metalloid concentrations. Coefficient of variation (based on 3 samples per taxa per site) across all sites for sediment metal concentrations and biotic concentrations.

Sample Type	As	Cd	Hg	MeHg	Pb	Se
Sediment	73.4	63.7	93.9	115.2	87.5	61.8
Amphipod	153.1	56.9	54.9	67.4	38.7	40.1
Crab	119.8	63.1	55.2	42.7	125.0	42.3
Fundulus	23.3	114.8	44.2	48.7	85.8	35.6
Mussel	60.7	71.1	64.1	42.3	59.2	19.5
Shrimp	43.5	65.1	37.3	51.1	92.2	39.6

**Table 3:** Relationships between biotic and sediment metal and metalloid concentrations. Summary of ANCOVA analyses for relationships between metals in biota, BSAFs, sediment concentrations, TOC-normalized sediment concentrations, TOC, and SEM.

Response Variable (Metal in Biota)	Sediment Characteristic	*R*^2^	Full Model *p*-Value	*p*-Value for Difference across Species	Test for Relationship between Metal Concentration in Biota and Sediment Characteristic	
					Slope ± SE	*p*-Value
As	Sed. Conc	40%	0.02	0.01	−0.01 ± 0.17	0.96
Cd	Sed. Conc	60%	<0.001	<0.001	0.16 ± 0.22	0.49
Hg	Sed. Conc	56%	0.001	0.001	0.24 ± 0.12	0.08
MeHg	Sed. Conc	29%	0.11	0.07	0.04 ± 0.1	0.72
Pb	Sed. Conc	42%	0.02	0.02	0.22 ± 0.14	0.17
Se	Sed. Conc	55%	0.001	0.001	−0.07 ± 0.13	0.62
As	TOC-corrected Sed. Conc.	43%	0.01	0.01	−0.36 ± 0.32	0.3
Cd	TOC-corrected Sed. Conc.	61%	<0.001	<0.001	0.69 ± 0.59	0.28
Hg **	TOC-corrected Sed. Conc.	64%	<0.001	<0.001	0.52 ± 0.16	0.02
MeHg	TOC-corrected Sed. Conc.	30%	0.11	0.07	0.07 ± 0.14	0.64
Pb	TOC-corrected Sed. Conc.	37%	0.04	0.02	0.2 ± 0.3	0.54
Se	TOC-corrected Sed. Conc.	55%	0.001	0.001	−0.04 ± 0.18	0.84
Cd	SEM Cd	62%	<0.001	<0.001	0.26 ± 0.18	0.21
Hg	SEM Hg	49%	0.004	0.002	0.05 ± 0.08	0.53
Pb **	SEM Pb	47%	0.006	0.01	0.5 ± 0.22	0.06
AsBSAF **	TOC	44%	0.01	0.09	−0.27 ± 0.09	0.02
CdBSAF **	TOC	66%	<0.001	<0.001	−0.27 ± 0.07	0.01
HgBSAF **	TOC	77%	<0.001	0.001	−0.28 ± 0.04	<0.001
MeHgBSAF **	TOC	54%	0.001	0.42	−0.36 ± 0.07	0.003
PbBSAF **	TOC	52%	0.002	0.06	−0.3 ± 0.08	0.01
SeBSAF **	TOC	54%	0.002	0.04	−0.2 ± 0.05	0.01
AsBSAF **	SEM-AVS	39%	0.03	0.11	0.02 ± 0.01	0.04
CdBSAF	SEM-AVS	51%	0.003	0.002	0.01 ± 0.01	0.23
HgBSAF **	SEM-AVS	70%	<0.001	0.002	0.03 ± 0.005	0.001
MeHgBSAF **	SEM-AVS	68%	<0.001	0.24	0.04 ± 0.01	0.001
PbBSAF	SEM-AVS	26%	0.17	0.17	0.01 ± 0.01	0.25
SeBSAF **	SEM-AVS	44%	0.01	0.07	0.02 ± 0.01	0.03

Cases where the predictor effect was statistically significant are noted with **.

**Table 4:** Summary of General Linear Model (GLM) analyses for relationships between metals in biota and stable isotope signatures (^13^C and ^15^N) across sites. Lower ^13^C indicates more pelagic feeding; higher ^15^N indicates higher trophic level.

Response Variable (Metal in Biota)	*R*^2^	Full Model *p*-Value	*p*-Value for Difference across Sites	Test for Relationship between Metal Concentration and Pelagic Feeding (^13^C)		Test for Relationship between Metal Concentration and Trophic Level (^15^N)	

				^13^C *p*-Value	^13^C Slope ± SE	^15^N *p*-Value	^15^N Slope ± SE
As	31%	0.24	0.14	0.09	0.08 ± 0.04	0.03	−0.11 ± 0.05
Cd	35%	0.16	0.37	0.18	−0.08 ± 0.06	0.29	−0.07 ± 0.06
Hg **	58%	<0.001	0.03	<0.001	−0.09 ± 0.02	0.24	0.03 ± 0.03
MeHg **	66%	<0.001	<0.001	<0.001	−0.08 ± 0.02	0.02	0.06 ± 0.02
Pb	43%	0.05	0.15	0.32	−0.04 ± 0.04	0.13	−0.07 ± 0.04
Se	15%	0.77	0.99	0.14	−0.04 ± 0.03	0.88	0 ± 0.03
%MeHg **	41%	0.07	0.41	0.95	−0.08 ± 1.46	0.01	4.39 ± 1.66

Cases where the predictor effect was statistically significant are noted with **.

## Abbreviations

The following abbreviations are used in this manuscript:

SEM-AVS: Simultaneously Extracted Metal-Acid Volatile Sulfides
BSAF: Benthic-Sediment Accumulation Factor
TOC: Total Organic Carbon

## References

[1] Agency for Toxic Substances and Disease Registry. Comprehensive Environmental Response, Compensation, and Liability Act Priority List Of Hazardous Substances.

[2] Monperrus M, Point D, Grall J, Chauvaud L, Amouroux D, Bareille G, Donard O (2005). Determination of metal and organometal trophic bioaccumulation in the benthic macrofauna of the Adour estuary coastal zone (SW France, Bay of Biscay). J. Environ. Monit.

[3] McKinley AC, Dafforn KA, Taylor MD, Johnston EL (2011). High levels of sediment contamination have little influence on estuarine beach fish communities. PLoS ONE.

[4] Langston WJ, O’Hara S, Pope ND, Davey M, Shortridge E, Imamura M, Harino H, Kim A, Vane CH (2012). Bioaccumulation surveillance in milford haven waterway. Environ. Monit. Assess.

[5] Rainbow PS, Kriefman S, Smith BD, Luoma SN (2011). Have the bioavailabilities of trace metals to a suite of biomonitors changed over three decades in sw england estuaries historically affected by mining?. Sci. Total Environ.

[6] National Research Council (2003). Bioavailability of Contaminants in Soils and Sediments: Processes, Tools, and Applications.

[7] Luoma SN, Rainbow PS (2008). Metal Contamination in Aquatic Environments: Science and Lateral Management.

[8] Mathews T, Fisher NS (2008). Evaluating the trophic transfer of cadmium, polonium, and methylmercury in an estuarine food chain. Environ. Toxicol. Chem.

[9] Langston WJ (1982). The distribution of mercury in british estuarine sediments and its availability to deposit-feeding bivalves. J. Mar. Biol. Assoc. UK.

[10] Chapman PM, Wang FY, Janssen CR, Goulet RR, Kamunde CN (2003). Conducting ecological risk assessments of inorganic metals and metalloids: Current status. Hum. Ecol. Risk Assess.

[11] Langston WJ, Zhou MJ (1987). Cadmium accumulation, distribution and metabolism in the gastropod littorina-littorea—The role of metal-binding proteins. J. Mar. Biol. Assoc. UK.

[12] Bryan GW, Langston WJ (1992). Bioavailability, accumulation and effects of heavy-metals in sediments with special reference to united-kingdom estuaries—A review. Environ. Pollut.

[13] Wang WX (2002). Interactions of trace metals and different marine food chains. Mar. Ecol. Prog. Ser.

[14] Wang WX, Rainbow PS (2005). Influence of metal exposure history on trace metal uptake and accumulation by marine invertebrates. Ecotoxicol. Environ. Saf.

[15] Ankley GT (1996). Evaluation of metal/acid-volatile sulfide relationships in the prediction of metal bioaccumulation by benthic macroinvertebrates. Environ. Toxicol. Chem.

[16] Borgmann U, Norwood WP (2002). Metal bioavailability and toxicity through a sediment core. Environ. Pollut.

[17] Borgmann U, Norwood WP, Dixon DG (2004). Re-evaluation of metal bioaccumulation and chronic toxicity in hyalella azteca using saturation curves and the biotic ligand model. Environ. Pollut.

[18] Marsden ID, Rainbow PS (2004). Does the accumulation of trace metals in crustaceans affect their ecology—The amphipod example?. J. Exp. Mar. Biol. Ecol.

[19] Lee JS, Lee JH (2005). Influence of acid volatile sulfides and simultaneously extracted metals on the bioavailability and toxicity of a mixture of sediment-associated Cd, Ni, and Zn to polychaetes neanthes arenaceodentata. Sci. Total Environ.

[20] Taylor DL, Linehan JC, Murray DW, Prell WL (2012). Indicators of sediment and biotic mercury contamination in a southern new england estuary. Mar. Pollut. Bull.

[21] Kim E, Kim H, Shin KH, Kim MS, Kundu SR, Lee BG, Han S (2012). Biomagnification of mercury through the benthic food webs of a temperate estuary: Masan bay, korea. Environ. Toxicol. Chem.

[22] Jones HJ, Swadling KM, Bulter ECV, Macleod CK (2014). Complex patterns in fish e sediment mercury concentrations in a contaminated estuary: The influence of selenium co-contamination?. Estuar. Coast. Shelf Sci.

[23] Michiels C, Raes M, Toussaint O, Remacle J (1994). Importance of se-glutathione peroxidase, catalase, and Cu/Zn-sod for cell-survival against oxidative stress. Free Radic. Biol. Med.

[24] Hamilton SJ (2004). Review of selenium toxicity in the aquatic food chain. Sci. Total Environ.

[25] Rainbow PS (2002). Trace metal concentrations in aquatic invertebrates: Why and so what?. Environ. Pollut.

[26] Greenwood NN, Earnshaw A (1984). Chemistry of the Elements.

[27] Benjamin MM, Leckie JO (1982). Effects of complexation by chloride, sulfate, and thiosulfate on adsorption behavior of cadmium on oxide surfaces. Environ. Sci. Technol.

[28] Mathews T, Fisher NS (2008). Trophic transfer of seven trace metals in a four-step marine food chain. Mar. Ecol. Prog. Ser.

[29] Yang D-Y, Chen Y-W, Gunn JM, Belzile N (2008). Selenium and mercury in organisms: Interactions and mechanisms. Environ. Rev.

[30] Chen CY, Folt CL (2000). Bioaccumulation and diminution of arsenic and lead in a freshwater food web. Environ. Sci. Technol.

[31] Chen CY, Folt CL, Stemberger RS, Blum JD, Klaue B, Pickhardt PC (2000). Accumulation of heavy metals in food web components across a gradient of lakes. Limnol. Oceanogr.

[32] Dutton J, Fisher NS (2011). Bioaccumulation of, As, Cd, Cr, Hg(II), and mehg in killifish (Fundulus heteroclitus) from amphipod and worm prey. Sci. Total Environ.

[33] Deegan LA, Garritt RH (1997). Evidence for spatial variability in estuarine food webs. Mar. Ecol. Prog. Ser.

[34] Young T, Komarow S, Deegan L, Garritt R (1999). Population size and summer home range of the green crab, carcinus maenas, in salt marsh tidal creeks. Biol. Bull.

[35] Fry B, Cieri M, Hughes J, Tobias C, Deegan LA, Peterson B (2008). Stable isotope monitoring of benthic-planktonic coupling using salt marsh fish. Mar. Ecol. Prog. Ser.

[36] Jones SH (2000). A Technical Characterization of Estuarine and Coastal New Hampshire.

[37] Kahl JS, Nelson SJ, Fernandez I, Haines T, Norton S, Wiersma GB, Jacobson G, Amirbahman A, Johnson K, Schauffler M (2007). Watershed nitrogen and mercury geochemical fluxes integrate landscape factors in long-term research watersheds at Acadia national park, Maine, USA. Environ. Monit. Assess.

[38] Sunderland EM, Amirbahman A, Burgess NM, Dalziel J, Harding G, Jones SH, Kamai E, Karagas MR, Shi X, Chen CY (2012). Mercury sources and fate in the gulf of maine. Environ. Res.

[39] Chen CY, Dionne M, Mayes BM, Ward DM, Sturup S, Jackson BP (2009). Mercury bioavailability and bioaccumulation in estuarine food webs in the gulf of maine. Environ. Sci. Technol.

[40] Perna L, LaCroix-Fralish A, Sturup S (2005). Determination of inorganic mercury and methylmercury in zooplankton and fish samples by speciated isotopic dilution GC-ICP-MS after alkaline digestion. J. Anal. At. Spectrom.

[41] Peterson BJ, Fry B (1987). Stable isotopes in ecosystem studies. Ann. Rev. Ecol. Syst.

[42] Sullivan MJ, Moncreiff CA (1990). Edaphic algae are an important component of salt-marsh food-webs—Evidence from multiple stable isotope analyses. Mar. Ecol. Prog. Ser.

[43] Fisher NS, Teyssie JL, Fowler SW, Wang WX (1996). Accumulation and retention of metals in mussels from food and water: A comparison under field and laboratory conditions. Environ. Sci. Technol.

[44] Stribling JM, Cornwell JC (1997). Identification of important primary producers in a chesapeake bay tidal creek system using stable isotopes of carbon and sulfur. Estuaries.

[45] Monikh FA, Safahieh A, Savari A, Doraghi A (2013). Heavy metal concentration in sediment, benthic, benthopelagic, and pelagic fish species from Musa Estuary (Persian Gulf). Environ. Monit. Assess.

[46] Chen CY, Borsuk ME, Bugge DM, Hollweg T, Balcom PH, Ward DM, Williams J, Mason RP (2014). Benthic and pelagic pathways of methylmercury bioaccumulation in estuarine food webs of the northeast United States. PLoS ONE.

[47] Amiard JC, Geffard A, Amiard-Triquet C, Crouzet C (2007). Relationship between the lability of sediment-bound metals (Cd, Cu, Zn) and their bioaccumulation in benthic invertebrates. Estuar. Coast. Shelf Sci.

[48] Baumann Z, Fisher NS (2011). Relating the sediment phase speciation of arsenic, cadmium, and chromium with their bioavailability for the deposit-feeding polychaete nereis succinea. Environ. Toxicol. Chem.

[49] Sotos-Jiménez MF, Arellano-Fiore C, Rocha-Velarde R, Jara-Marini ME, Ruelas-Inzunza J, Páez-Osuna F (2011). Trophic transfer of lead through a model marine four-level food chain: Tetraselmis suecica, Artemia franscscana, Litopenaeus vannamei, and Haemulon scudderi. Arch. Environ. Contam. Toxicol.

[50] Ray S, McLeese D, Pezzack D (1980). Accumulation of cadmium by nereis-virens. Arch. Environ. Contam. Toxicol.

[51] Metian M, Warnau M, Oberhansli F, Bustamante P (2009). Delineation of Pb contamination pathways in two pectinidae: The variegated scallop *Chlamys varia* and the king scallop *Pecten maximus*. Sci. Total Environ.

[52] Bryan GW (1985). The use of multi-estuary comparisons to elucidate factors governing the bioaccumulation and effects of heavy-metals in benthic organisms. Estuaries.

[53] Fisher NS, Reinfelder JR, Tessier A, Turner DR (1995). The trophic transfer of metals in marine systems. Metal Speciation and Bioavailability in Aquatic Systems.

[54] Harris RC, Bodaly RA (1998). Temperature, growth and dietary effects on fish mercury dynamics in two Ontario lakes. Biogeochemistry.

[55] Mason RP, Laporte JM, Andres S (2000). Factors controlling the bioaccumulation of mercury, methylmercury, arsenic, selenium, and cadmium by freshwater invertebrates and fish. Arch. Environ. Contam. Toxicol.

[56] Chen C, Amirbahman A, Fisher N, Harding G, Lamborg C, Nacci D, Taylor D (2008). Methylmercury in marine ecosystems: Spatial patterns and processes of production, bioaccumulation, and biomagnification. EcoHealth.

[57] Adams WJ, Blust R, Borgmann U, Brix KV, DeForest DK, Green AS, Meyer JS, McGeer JC, Paquin PR, Rainbow PS (2010). Utility of tissue residues for predicting effects of metals on aquatic organisms. Integr. Environ. Assess. Manag.

[58] Berry WJ, Hansen DJ, Mahony JD, Robson DL, DiToro DM, Shipley BP, Rogers B, Corbin JM, Boothman WS (1996). Predicting the toxicity of metal-spiked laboratory sediments using acid-volatile sulfide and interstitial water normalizations. Environ. Toxicol. Chem.

[59] US Environmental Protection Agency (2005). Procedures for the Derivation of Equilibrium Partitioning Sediment Benchmarks (ESBX) for the Protection of Benthic Organisms: Metal Mixtures (Cadmium, Copper, Lead, Nickel, Silver and Zinc).

[60] Lee BG, Griscom SB, Lee JS, Choi HJ, Koh CH, Luoma SN, Fisher NS (2000). Influences of dietary uptake and reactive sulfides on metal bioavailability from aquatic sediments. Science.

[61] De Jonge M, Dreesen F, de Paepe J, Blust R, Bervoets L (2009). Do acid volatile sulfides (AVS) influence the accumulation of sediment-bound metals to benthic invertebrates under natural field conditions?. Environ. Sci. Technol.

[62] Wang WX, Fisher NS (1999). Assimilation efficiencies of chemical contaminants in aquatic invertebrates: A synthesis. Environ. Toxicol. Chem.

[63] Hare L, Tessier A, Borgmann U (2003). Metal sources for freshwater invertebrates: Pertinence for risk assessment. Hum. Ecol. Risk Assess.

[64] Balcom PH, Schartup AT, Mason RP, Chen CY (2015). Sources of water column methylmercury across multiple estuaries in the Northeast U.S. Mar. Chem.

[65] Power M, Klein GM, Guiguer K, Kwan MKH (2002). Mercury accumulation in the fish community of a sub-Arctic lake in relation to trophic position and carbon sources. J. Appl. Ecol.

[66] Campbell LM, Norstrom RJ, Hobson KA, Muir DCG, Backus S, Fisk AT (2005). Mercury and other trace elements in a pelagic Arctic marine food web (Northwater Polynya, Baffin Bay). Sci. Total Environ.

[67] Hammerschmidt CR, Fitzgerald WF (2006). Bioaccumulation and trophic transfer of methylmercury in long island sound. Arch. Environ. Contam. Toxicol.

[68] Driscoll CT, Han YJ, Chen CY, Evers DC, Lambert KF, Holsen TM, Kamman NC, Munson RK (2007). Mercury Contamination in Forest and Freshwater Ecosystems in the Northeastern United States. Bioscience.

[69] Kidd KA, Muir DCG, Evans MS, Wang X, Whittle M, Swanson HK, Johnston T, Guildford S (2012). Biomagnification of mercury through lake trout (Salvelinus namaycush) food webs of lakes with different physical, chemical and biological characteristics. Sci. Total Environ.

[70] Lavoie RA, Jardine TD, Chumchal MM, Kidd KA, Campbell LM (2013). Biomagnification of mercury in aquatic food webs: A worldwide meta-analysis. Environ. Sci. Technol.

