# Supplementary material for: Metal Bioaccumulation by Estuarine Food Webs in New England, USA
Source: J Mar Sci Eng. Author manuscript; Available in PMC 2017 Jun 2. (PMC5455787; doi:10.3390/jmse4020041)
Supplement: Erratum [file NIHMS828554-supplement-Erratum.pdf]

Journal of
Marine Science and Engineering

MDPI

Erratum

Erratum: Chen, C.Y.; Ward, D.M.; Williams, J.J.; Fisher, N.S. Metal Bioaccumulation by Estuarine Food Webs in New England, USA. *J. Mar. Sci. Eng.* 2016, *4*, 41

Celia Y. Chen [1,*], Darren M. Ward [2], Jason J. Williams [3] and Nicholas S. Fisher [4]

1 Department of Biological Sciences, Dartmouth College, Hanover, NH 03755, USA
2 Department of Fisheries Biology, Humboldt State University, Arcata, CA 95521, USA; darren.ward@humboldt.edu
3 Department of Civil and Environmental Engineering, Washington State University, Pullman, WA 99164, USA; jason.williams2@email.wsu.edu
4 School of Marine and Atmospheric Sciences, Stony Brook University, Stony Brook, NY 11794, USA; nicholas.fisher@stonybrook.edu
* Correspondence: celia.chen@dartmouth.edu; Tel.: +1-603-646-2376

Received: 1 September 2016; Accepted: 2 September 2016; Published: 5 September 2016

The authors wish to correct the NIH grant number in this paper [1] as follows:

Acknowledgments: We are grateful to Brandon Mayes for conducting fieldwork and to Amy Higgins for gathering research materials and creating field site maps. We also thank Brian Jackson, Vivien Taylor and Arthur Baker of the Dartmouth Trace Element Analysis Core for metal analysis of all our samples. This research was funded by the U.S. Department of Defense Strategic Environmental Research and Development Program (SERDP) Project ER-1503 and by the National Institutes of Health Grant P42 ES007373 from the National Institute of Environmental Health Sciences.

Reference

1. Chen, C.Y.; Ward, D.M.; Williams, J.J.; Fisher, N.S. Metal Bioaccumulation by Estuarine Food Webs in New England, USA. *J. Mar. Sci. Eng.* **2016**, *4*, 41. [CrossRef]

© 2016 by the authors; licensee MDPI, Basel, Switzerland. This article is an open access article distributed under the terms and conditions of the Creative Commons Attribution (CC-BY) license (http://creativecommons.org/licenses/by/4.0/).
